# AFM Imaging of Protein Aggregation in Studying the Impact of Knotted Electromagnetic Field on A Peroxidase

**DOI:** 10.1038/s41598-020-65888-z

**Published:** 2020-06-02

**Authors:** Yuri D. Ivanov, Tatyana O. Pleshakova, Ivan D. Shumov, Andrey F. Kozlov, Irina A. Ivanova, Anastasia A. Valueva, Vadim Yu. Tatur, Mikhail V. Smelov, Nina D. Ivanova, Vadim S. Ziborov

**Affiliations:** 1 Institute of Biomedical Chemistry, Pogodinskaya str., 10, Moscow, 119121 Russia; 2 Foundation of Perspective Technologies and Novations, Moscow, 115682 Russia; 3 Skryabin Moscow State Academy of Veterinary Medicine and Biotechnology, Moscow, 109472 Russia; 4 Joint Institute for High Temperatures of the Russian Academy of Sciences, Moscow, 125412 Russia

**Keywords:** Oxidoreductases, Metalloproteins, Applications of AFM

## Abstract

The phenomenon of knotted electromagnetic field (KEMF) is now actively studied, as such fields are characterized by a nontrivial topology. The research in this field is mainly aimed at technical applications – for instance, the development of efficient communication systems. Until present, however, the influence of KEMF on biological objects (including enzyme systems) was not considered. Herein, we have studied the influence of KEMF on the aggregation and enzymatic activity of a protein with the example of horseradish peroxidase (HRP). The test HRP solution was irradiated in KEMF (the radiation power density was 10^−12^ W/cm^2^ at 2.3 GHz frequency) for 40 min. After the irradiation, the aggregation of HRP was examined by atomic force microscopy (AFM) at the single-molecule level. The enzymatic activity was monitored by conventional spectrophotometry. It has been demonstrated that an increased aggregation of HRP, adsorbed on the AFM substrate surface, was observed after irradiation of the protein sample in KEMF with low (10^−12^ W/cm^2^) radiation power density; at the same time, the enzymatic activity remained unchanged. The results obtained herein can be used in the development of models describing the interaction of enzymes with electromagnetic field. The obtained data can also be of importance considering possible pathological factors that can take place upon the influence of KEMF on biological objects— for instance, changes in hemodynamics due to increased protein aggregation are possible; the functionality of protein complexes can also be affected by aggregation of their protein subunits. These effects should also be taken into account in the development of novel highly sensitive systems for human serological diagnostics of breast cancer, prostate cancer, brain cancer and other oncological pathologies, and for diagnostics of diseases in animals, and crops.

## Introduction

It is known that electromagnetic radiation of various intensity can have different influence on human body. Electromagnetic fields can have various topology, such as transverse and knotted one (knotted electromagnetic field, KEMF)^1,2^. The simplest and most common electromagnetic waves are transverse ones, and, to date, their effects have been widely studied in various frequency and intensity ranges. As regards biomedical applications, microwave radiation is interesting in that, depending on its intensity, it is employed in biological research, and in both medical diagnostics and therapy. In this way, upon exposure of biological tissues to high-intensity radiation (~90 W/cm^2^), their temperature increases to ~90 °С, and denaturation of biological objects is observed. It was shown that, under such conditions, partial loss of functional activity of proteins (for instance, peroxidase) is observed^3^. At lower radiation intensity (10 μW/cm^2^ ^4^), both positive therapeutic effects (which usually take place owing to local heating^5^) and negative effects are observed. Here, it should be noted that studies on the application of non-thermal effects of low-power microwave radiation (10^−5^ to 10^−3^ W/cm^2^) in cancer therapy were recently reported^6^.

Enzyme systems play an important role in various metabolic processes. For this reason, studying the influence of microwave radiation on enzymes is an important task from the viewpoints of both fundamental and applied science. In this way, an effect of enhancement of erythrocyte membrane resistance upon the exposure of the cells to a radiation of a 1.5 μW power was found^7^. Furthermore, in the studies on the interaction of microwave radiation with proteins, an increase in the catalytic activity of alanine aminotransferase was observed^8^.

From a practical point of view, the microwave (from 2 to 4 GHz) frequency range is the most interesting one. A number of devices called radiometers are functioning in this range. These devices allow one to monitor the functional state of a human body in the event of pathologies^9^, to register the microwave radiation upon excitation of aqueous and protein media^10^, and also to monitor the functional activity of protein systems by registering changes in the brightness temperature^11,12^. The level of background radiation within a typical radiometer bandwidth (~0.1 GHz) at a temperature of 310 К makes up 4 × 10^−13^ W^13^, while pathologies in human body are accompanied by radiation in the same frequency range at a level corresponding to a change in the brightness temperature of the order of 0.4 °С^13^. In this connection, studies on the influence of electromagnetic radiation in these low-level ranges of power and frequencies on biological objects seem to be relevant.

At present, the theory considering electromagnetic fields with a different topology, known as «knotted electromagnetic fields» (KEMF), is developed; corresponding practical studies were reported^14–17^. These fields have a certain practical relevance, as they can be used in the industry of efficient new generation communication systems. These fields have a specific topology and can influence surrounding biological environment (including staff) — for instance, through the mechanism of changing the physicochemical properties of proteins (particularly enzymes). To date, however, this mechanism has not been studied.

For this reason, in our present work, we have studied the influence of KEMF with 2.3 GHz frequency and a power density of 10^−12^W/cm^2^ on an enzyme protein (with the example of horseradish peroxidase, HRP). The KEMF frequency and power have been selected based on the above-listed considerations: such parameters of the electromagnetic field are interesting from the practical point of view for monitoring pathologies in human and for studying enzymatic reactions. HRP protein is a well-studied enzyme and is widely used as a model object in studies of a wide class of peroxidases; this is why this protein has been used in our research. HRP pertains to heme-containing enzymes. Studying peroxidases is of great interest, since these enzymes are well represented in plant and animal tissues^18^ and play an important functional role in the organism. Peroxidase catalyzes the oxidation of a broad spectrum of organic and inorganic compounds by hydrogen peroxide^19^. For instance, myeloperoxidase plays an important role in human body by participating in atherogenesis^20^. The molecular weight of HRP is 40 kDa^21^. It is known that the molecules of many enzymes (including HRP) form aggregates^22^. Changes in the aggregation state of an enzyme under external physicochemical influence (such as thermal, chemical, electromagnetic etc.) characterize a change in its spatial structure. This can lead to pathologies in the organism. It is to be noted that, if the change in the structure does not affect the active site or chromophore groups of the enzyme, it is difficult to reveal such a change by monitoring the kinetic parameters of the catalysis reaction. For this reason, in our present research, atomic force microscopy (AFM) has been employed for studying the influence of electromagnetic field on the HRP aggregation. AFM allows one to perform visualization of protein aggregation and to measure the enzymatic activity at the level of single molecules of enzymes^23,24^. AFM has been used to determine the aggregation state of HRP before and after its exposure to KEMF. That is, the enzyme and its aggregates have been visualized on the surface of a solid substrate. In parallel, the enzymatic activity of HRP in solution has been determined by spectrophotometry.

It has been demonstrated that, after the exposure of HRP solution to KEMF, an increased aggregation of HRP macromolecules, adsorbed on the AFM substrate surface, is observed, while the functional activity of the protein remains unchanged. The results obtained herein can be used in the development of models describing the interaction of an electromagnetic field with either isolated enzyme systems or even whole organisms. Moreover, the obtained data are also important for further studies on the development of standards for working with electromagnetic radiation. Furthermore, since protein aggregation can occur in biosensor systems operating in the presence of external electromagnetic fields, the results obtained herein should be taken into account in the development of highly sensitive biosensors (which are sensitive to electromagnetic interference) – including nanowire-based biosensors intended for diagnostics of oncological diseases, such as breast cancer, prostate cancer, brain cancer etc.

## Results

### AFM visualization of HRP macromolecules

Firstly, to determine the optimal concentration of HRP solution, a series of experiments on AFM visualization of protein adsorbed onto mica substrates from HRP solution with a concentration from 10^−9^ M to 10^−6^ М has been performed. In this series, we analyzed the surface topography upon AFM visualization of the substrate surface after adsorption of HRP from the tested solutions onto mica substrates. Typical AFM images of mica surface with adsorbed HRP macromolecules are presented in Figures S1–S4. The results, obtained in this experimental series, are described in detail in the Supplementary Information. Briefly, compact objects, whose height was ≥1 nm, were attributed to the adsorbed biomolecules. One can point out that the number of these objects in the case with 10^−6^ M HRP solution is much greater than that in the case with 10^−7^ M one. At that, such objects were virtually not observed in the analysis of 10^−8^ M and 10^−9^ M HRP solutions. Thus, one can state the number of adsorbed HRP macromolecules to be dependent on the HRP solution concentration. Based on these results, the optimal concentration of HRP for the AFM experiments was determined to be 10^−7^ M (i.e. 0.1 µM). Accordingly, the AFM experiments on the determination of the effect of KEMF on HRP aggregation state were performed with 0.1 µM HRP solution.

**Figure 1:**
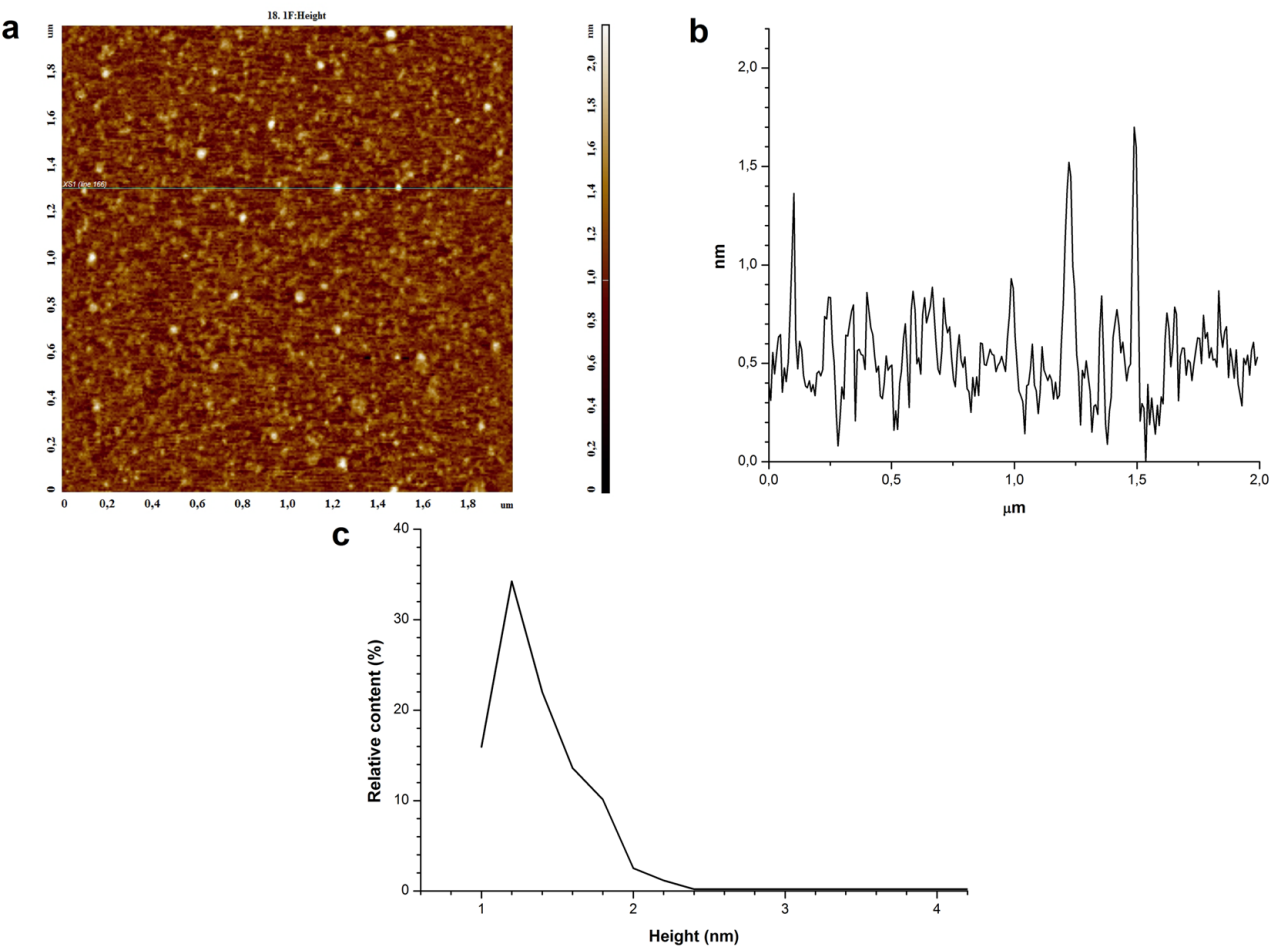
Results of control experiment: AFM analysis data obtained for 0.1 µM HRP solution, which was not exposed to KEMF. (**а**) Typical AFM image of mica surface with adsorbed HRP macromolecules. (**b**) Profile of a cross-section corresponding to the line in the image in **a**. (**с**) Density function plot *ρ(h)* obtained for the visualized objects.

**Figure 2:**
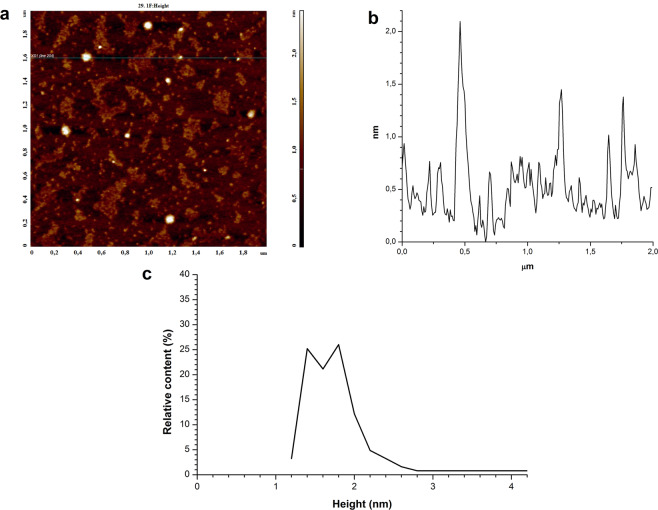
Results of AFM analysis of 0.1 µM HRP solution after its irradiation in KEMF. (**а**) Typical AFM image of mica surface with adsorbed HRP macromolecules. (**b**) Profile of a cross-section corresponding to the line in the image in **a**. (**с**) Density function plot *ρ(h)* obtained for the visualized objects.

Figures 1 and 2 display typical height AFM images of mica surface with HRP macromolecules adsorbed from the 0.1 µM solutions, which were either not exposed to KEMF (control experiment; Fig. 1) or irradiated in KEMF (Fig. 2). AFM allows one to measure both height and lateral dimensions of the studied macromolecules. It should be emphasized that just the macromolecules’ height can be correctly determined by AFM in tapping mode, as was reported in many previous studies^25^. At the same time, lateral dimensions of the AFM images of the studied objects depend on the curvature radius of the AFM probe. For this reason, the height of the AFM images is commonly used as the main parameter in the analysis of protein aggregation, and this is the approach we have used in our study.

As seen from Fig. 1(a), in the case when the protein solution was not exposed to the electromagnetic field, the protein adsorbed onto mica surface in the form of compact objects with height from 1.0 to 2.0 nm. After processing the obtained AFM data, the distribution of the number of the visualized objects with height (density function) *ρ(h)* was plotted. The corresponding density function plot is presented in Fig. 1(c). This plot indicates that the maximum of the *ρ(h)* distribution is equal to the value of (1.2 ± 0.2) nm, while the distribution width at half-height makes up 0.5 nm. According to Davies *et al*., the molecular weight of HRP is *M*_*r*_ = 40 kDa^21^. Furthermore, other globular proteins with similar *M*_*r*_ were reported to have comparable sizes (putidaredoxin reductase, *h*_*max*_ = 1.8 nm^26^; adrenodoxin reductase, *h*_*max*_ = 1.8 nm^27^, *M*_*r*_ = 54 kDa^28^). For these considerations, one can conclude that the objects with ~1.2-nm height, observed upon AFM scanning, can be attributed to HRP monomers. At the same time, the density function has an inflection point near 1.6 nm, what indicates nonmonotonic decrease of the *ρ(h)* curve in this height range. This indicates that the aggregate objects contribute to the right wing of the distribution, in the range of heights *h* ≥ 1.6 nm. Thus, we have observed the presence of HRP on the mica surface in the form of a mixture of monomers and aggregates.

As seen from Fig. 2(a), the shape of HRP macromolecules visualized by AFM has changed after the exposure of HRP solution to KEMF. Objects with heights from 1 to 2.5 nm are observed in the AFM image of mica surface with adsorbed protein macromolecules. As seen from the plot in Fig. 2(c), objects with heights greater than 1.6 nm make much more significant contribution to the distribution of the visualized objects with height, in comparison with the case without the exposure to KEMF shown in Fig. 1(c). This indicates that an increase in lateral sizes and in heights of the AFM visualized objects is observed after irradiation of protein solution in KEMF. Accordingly, one can conclude that aggregation of HRP enzyme is observed in the solution after its exposure to KEMF.

### HRP enzymatic activity measurements

**Figure 3:**
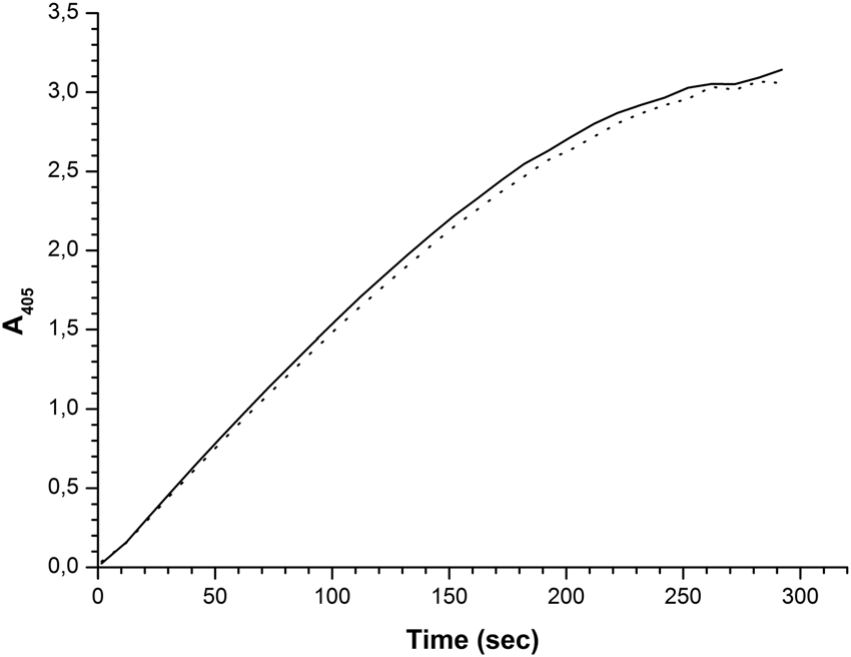
Spectrophotometric measurements of enzymatic activity of HRP. Characteristic time dependencies of change in solution absorbance at 405 nm obtained for control HRP sample (dotted line) and for HRP sample exposed to KEMF with 2.3 GHz frequency at 10^-12^ W/cm^2^ radiation power density for 40 min (solid line). Experimental conditions: HRP:ABTS:H_2_O_2_ = 10^-9^ M:3 mM:2.5 mM. T = 22 °C.

Spectrophotometric measurements of enzymatic activity of HRP were performed using ABTS substrate, as described in Materials and Methods, for both the control HRP solution and the solution irradiated in KEMF. Figure 3 displays characteristic time dependencies of change in solution absorbance at 405 nm. The curves in this Figure clearly indicate that the enzymatic activity of HRP did not change after the exposure of its solution to KEMF, since the curves obtained for the control solution and for the KEMF-irradiated one have virtually the same character.

## Discussion

The problem of the effect of microwave radiation on enzymes is of great interest. Particularly, this concerns the power range at the level of background radiation (~10^−12^ W/cm^2^), at which the radiation in enzyme systems is registered^29^ and pathological changes in human are detected^30^. The impact of microwave radiation on proteins is determined by both the radiation power and the exposure time. Thermal effect of electromagnetic radiation is determined by the energy that enters the system, leading to a significant (>0.1 °C) increase in its temperature^31,32^. Non-thermal effect occurs in the case if the energy received does not lead to such an increase in temperature^31^. After a 30-min exposure of a human body to a radiation with the power of 5 mW/cm^2^, its temperature increases by only 0.1 °C^31^. Taking into account that the human body consists mainly of water, the power range of ≤5 mW/cm^2^ is attributed to the power range of non-thermal effects of radiation on aqueous solutions. However, at such (and even lesser) power values, a significant impact of electromagnetic fields on the properties of proteins can be observed. For instance, after exposure to such fields, an increase in catalytic activity of alanine aminotransferase was observed, while no change in the activity of amylase was detected^8^. The occurrence of such effects is explained in the literature by various types of resonant interactions of radiation with biological objects^6^.

In the case of enzymes, the impact of microwave radiation on their physicochemical properties is traditionally estimated by measuring the change in their enzymatic activity after the exposure to the electromagnetic field^8^. This approach to the estimation of the impact of electromagnetic radiation on enzyme properties is, however, not fully faithful, since upon irradiation of the enzyme, the protein structure in its active site may not change, while changes in other, more labile, region of the protein globule are possible. This can lead to a change in the aggregation state of the protein. The latter assumption is confirmed by the results of our present study. In this way, we have observed changes in the aggregation state of HRP after the irradiation of its solution, while no change in its enzymatic activity has been registered. This indicates that the exposure to KEMF influences only the spatial structures of the protein, but does not affect its active site.

In our present work, the effect of KEMF, with a radiation power at the level slightly higher than that of the background, on the properties of HRP enzyme has been studied. This radiation has a different spatial topography than the commonly used transversely polarized one.

It should be noted that experiments for comparison of the influence of standard transverse electromagnetic field with that of knotted electromagnetic field on HRP have been performed as controls. Normal level of electromagnetic radiation, in its turn, includes electromagnetic radiation from industrial sources. We have conducted our experiments in the presence of such a background.

We exposed HRP solutions to a transverse electromagnetic radiation from a standard source (18 W BASIC T8 luminescent lamp, G13 base, L 18 W/765, OSRAM, Germany). The irradiation time was 40 min — as in the experiments with KEMF. The luminescent lamp used emits not only in visible, but also in the studied microwave range. The HRP solution to be irradiated was placed at such a distance from the lamp that the power of the microwave radiation with a frequency from 2.12 to 2.68 GHz was ~10^−8^ W/cm^2^ — i.e. much higher than that of KEMF employed in our experiments. After a 40-min irradiation, the HRP solution was studied by AFM, as in the case with KEMF. In this way, we have not revealed any effect of the radiation from the luminescent lamp on HRP aggregation, despite the microwave radiation power from the lamp was much higher than that in the case of KEMF. Thus, against the background of the normal level of electromagnetic radiation from the environment, a change in the aggregation state of the protein is only observed in the field with a knotted topology, but not in the field with standard transverse topology. The matter of such a different influence of fields with different topology on the enzyme aggregation has not yet been studied. The possible explanation of this phenomenon can be as follows. Since protein molecules are chiral, their response to the influence of electromagnetic field consists in their polarization. It is known that in the GHz frequency range, the dielectric constant of a biological medium depends on the direction of normal line to the front of an electromagnetic wave^33^. Thus, with assumption that these considerations are correct for an aqueous protein solution, different response from protein fragments is expected. One should also take into account that the protein molecules are hydrated^34^. Furthermore, water has a complex structure and represents a mixture of ortho- and para- isomers. So, protein hydration leads to a shift in the ortho/para ratio, causing selective binding of para-H_2_O upon formation of hydration shells on biomolecules^35^. In the microwave range there are resonances of the ortho- and para- isomers of water^35,36^. Owing to a concentration of force lines along the selected direction of the propagation axis of electromagnetic radiation, knotted fields possibly cause more significant impact on the structure of the hydration shell of the protein, leading to a more complete ortho/para conversion of water and providing stronger polarization of protein molecules — in comparison with transverse fields. At that, the active site of the enzyme is not significantly affected. Hydration shells hinder enzyme coagulation — as was observed, for instance, for hemoglobin^37^. As a result of partial destruction of the hydration shell, the structure of the surface layer of the enzyme molecule becomes more labile, and this leads to an increased aggregation of the enzyme on mica surface after the exposure to KEMF.

Changes in the aggregation state of proteins are critical upon the participation of individual biomolecules in the formation of functional multi-protein complexes. In this way, for instance, it is known that myeloperxidase in the form of dimers can participate in the formation of ternary seroplasmin/lactoferrin/myeloperoxidase complexes, which regulate inflammation process^38^. Accordingly, the change in the aggregation state of myeloperxidase will lead to a change in the composition of these complexes. Moreover, protein aggregation can hinder hemodynamics in small vessels of the body.

Thus, the development of relevant approaches for the evaluation of effects of radiation on the properties of enzymes and other proteins is required. Monitoring of the effect of low-intensity disturbing microwave radiation on the human body is also necessary. Account for this effect is also of use in the development of new-generation biosensors (operating in the presence of external electromagnetic fields), whose high sensitivity allows them to be employed for early diagnosis of diseases, such as breast cancer, prostate cancer, brain cancer, which pertain to the most aggressive oncological diseases.

## Conclusions

The impact of knotted electromagnetic field (KEMF) on the aggregation state of horseradish peroxidase (HRP) enzyme in aqueous solution has been studied. It has been demonstrated that this radiation, at low (10^−12^W/cm^2^) power density, leads to an increased aggregation of the enzyme on mica surface after the exposure of HRP to KEMF. At that, the enzymatic activity of HRP remains unchanged. The results obtained herein are of importance for studying the influence of KEMF on human body, and also for the development of shielding systems and standards regulating the use of this radiation.

## Methods

### Chemicals and protein

Peroxidase from horseradish HRP was purchased from Sigma (St. Louis, MO, USA). 2,2′-azino-bis(3-ethylbenzothiazoline-6-sulfonate) (ABTS) was purchased from Sigma. Disodium hydrogen orthophosphate (Na_2_HPO_4_), citric acid and hydrogen peroxide (H_2_O_2_) were purchased from Reakhim (Moscow, Russia). All solutions were prepared using deionized ultrapure water (with 18.2 MΩ×cm resistivity) obtained with a Simplicity UV system (Millipore, Molsheim, France).

### HRP enzymatic activity assay

HRP activity was estimated according to the technique described in detail by Sanders *et al*.^39^ employing ABTS as reducing substrate. Briefly, the rate of change in solution absorbance at 405 nm was measured employing an Agilent 8453 UV-visible spectrophotometer (Agilent Technologies Deutschland GmbH, Waldbronn, Germany). 30 μL of 10^−7^ M HRP solution were added into a 3-mL quartz cuvette (pathlength 1 cm, Agilent, USA) containing 2.96 mL of 0.3 mM ABTS solution in phosphate-citrate buffer (51 mM Na_2_HPO_4_, 24 mM citric acid, pH 5.0) and stirred. In this way, final HRP concentration in the cuvette was 10^−9^ M. Finally, 8.5 μL of 3% (w/w) H_2_O_2_ were added into the cuvette. Spectrum acquisition was started immediately after the addition of H_2_O_2_. All samples were analyzed in at least three technical replicates.

### Experimental setup for protein solution irradiation

In the papers by Rañada^2,14,40,41^, new topologically nontrivial solutions of Maxwell equations in vacuum, based on the method of Hopf bundles of the physical fields space, were described. These solutions are presented in the form of knotted magnetic and electric force lines. Importantly, these lines do not self-intersect and are described by regular functions. They have not only a curvature, but also a torsion; the latter determines the new degree of freedom of the electromagnetic field. The curvature of the force lines allows their closing on a torus into a topological knot — for instance, into a “five-leafed sheet”. KEMF generation is performed with a knotted antenna, in which the number of knotted wire loops determines that of knots in the electromagnetic field.

In the paper by Werner *et al*.^42^, theoretical data on the calculation of the directional patterns of knotted antennas, which generate KEMF, were given. Smelov *et al*.^16,17^ considered the design of knotted antennas generating KEMF. Knotted antenna represents a five-leafed wire knot. To match the transmitting and receiving antennas, the transmitting antenna is fabricated with right winding (right helicity), while the receiving antenna is fabricated with left winding (left helicity). The central rod, on which the five-leafed antenna is located, is connected to a half-wave vibrator with balancing and matching U-knee using a 50-Ω cable.

As a consequence, the force lines of the so-generated electromagnetic field are knotted, but do not self-intersect, while the wave front has a helical shape. Electric force lines are twisted into a topological braid along the wave vector and the radius vector on the five-leaf sheet’s axis.

**Figure 4:**
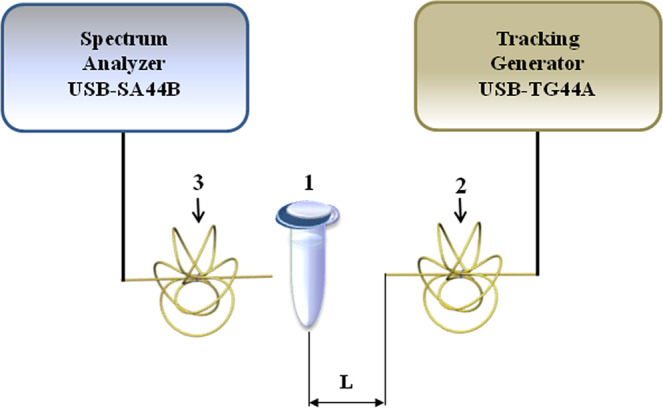
Experimental setup. Numbers indicate the tube with a protein sample (1), the USB-TG44A tracking generator with a five-leafed wire knot antenna (2), the USB-SA44B spectrum analyzer with a five-leafed wire knot antenna (3); *L* is the distance between the antenna and the tube.

The experimental setup, used for studying the influence of knotted (KEMF) radiation on a protein solution, is schematically represented in Fig. 4. An Eppendorf-type polypropylene tube, containing 1 mL of protein solution, was placed at a certain distance (*L*) from the KEMF emitter (antenna). The electromagnrtic field parameters (radiation power density of 10^−12^ W/cm^2^ at 2.3 GHz frequency) were set with a USB-TG44A Tracking Generator (Signal Hound, USA). The radiation power was controlled with a USB-SA44B Spectrum Analyzer (Signal Hound, USA). The distance between the emitter and the tube was *L* = 64 cm. The protein solution was irradiated in KEMF for 40 min. The temperature of the enzyme solution has been measured within the studied ranges of radiation energy and irradiation time. It has been demonstrated that, under the conditions of our experiments, the solution temperature did not change significantly, and the magnitude of the changes was no greater than 0.2 °C. After the irradiation, the protein solution was analyzed by AFM (to estimate the aggregation state of HRP) and by spectrophotometry (to perform comparative estimation of its enzymatic activity).

### AFM experiments and sample preparation

The AFM experiments were carried out using the direct surface adsorption method^25^. Muscovite mica sheets (SPI, USA) were used as AFM substrates with hydrophilic surface. For AFM sample preparation, a freshly cleaved mica sheet was immersed into 800 µL of 0.1 μM solution of HRP protein in deionized ultrapure water, which was either irradiated or not irradiated (control experiment) in KEMF. The AFM substrate was incubated in the protein solution for 10 min at room temperature in a shaker at 600 rpm. During the incubation, the protein macromolecules adsorbed onto the mica surface. After the incubation, each substrate was rinsed with ultrapure water and dried in air.

The protein concentration used in AFM experiments was selected according to the results of preliminary experimental series on the AFM visualization of HRP adsorbed onto mica substrates from the protein solutions with a concentration ranging from 10^−9^ M to 10^−6^ М. Results obtained in this experimental series are described in the Supplementary Information.

Mica surface with adsorbed protein macromolecules was visualized by AFM. This method allows one to reliably measure the heights of single macromolecules with high (0.1 nm) resolution^25,43^. At the same time, lateral sizes of the resulting AFM images of the studied macromolecules can exceed their real sizes due to the effect of the AFM cantilever’s curvature radius (i.e., the effect of convolution of the probe and the objects under study)^25,43^. For this reason, in our present study, only the height of AFM images was used as a criterion for the determination of an increase in HRP macromolecules’ sizes (i.e., for the determination of HRP aggregation). All AFM measurements were carried out in tapping mode in air employing a Titanium multimode atomic force microscope (NT-MDT, Russia; this equipment pertains to the equipment of “Human Proteome” Core Facility of the Institute of Biomedical Chemistry, supported by Ministry of Education and Science of Russian Federation, agreement 14.621.21.0017, unique project ID RFMEFI62117X0017) with NSG10 cantilevers (“TipsNano”, Zelenograd, Russia; from 140 to 390 kHz resonant frequency, from 3.1 to 37.6 N/m force constant, tip curvature radius <10 nm). The calibration of the microscope by height was carried out on a *TGZ1* calibration grating (NT-MDT, Russia; step height 21.4 ± 1.5 nm). The total number of imaged objects in each sample was no less than 200, and the number of frames for each sample was no less than 10. All samples were analyzed in at least three technical replicates. The density of the distribution of the AFM visualized objects with height *ρ(h)* was calculated as *ρ(h)* = *(N*_*h*_/N)*100%, where *N*_*h*_ is the number of imaged proteins with height *h*, and *N* is the total number of imaged proteins. Control experiments were performed with use of protein-free ultrapure water instead of protein solution; in these experiments, no objects with >0.5 nm height were registered.

AFM operation, obtaining AFM images, their treatment (flattening correction etc.) and exporting the obtained data in ASCII format were performed using a standard NOVA Px software (NT-MDT, Moscow, Zelenograd, Russia) supplied with the atomic force microscope.

The number of the visualized particles in the obtained AFM images was calculated automatically using a specialized AFM data processing software developed in Institute of Biomedical Chemistry (Rospatent registration no. 2010613458).

## Supplementary information

Results of AFM visualization of protein adsorbed onto mica substrates from HRP solution with a concentration from 10<sup>-9</sup> to 10<sup>-6</sup> М.

## Data Availability

Correspondence and requests for materials should be addressed to Yu.D.I.
