# Supplementary material for: AFM Imaging of Protein Aggregation in Studying the Impact of Knotted Electromagnetic Field on A Peroxidase
Source: Sci Rep. 2020 Jun 2;10:9022. doi: 10.1038/s41598-020-65888-z (PMC7265551; doi:10.1038/s41598-020-65888-z)
Supplement: Supplementary file 1 — Results of AFM visualization of protein adsorbed onto mica substrates from HRP solution with a concentration from 10-9 to 10-6 М. [file 41598_2020_65888_MOESM1_ESM.docx]

SUPPLEMENTARY INFORMATION

*AFM IMAGING OF PROTEIN AGGREGATION IN STUDYING THE IMPACT OF KNOTTED ELECTROMAGNETIC FIELD ON A PEROXIDASE*

Yuri D. Ivanov,^1, *^ Tatyana O. Pleshakova,^1^ Ivan D. Shumov,^1^ Andrey F. Kozlov,^1^ Irina A. Ivanova,^1^ Anastasia A. Valueva,^1^ Vadim Yu. Tatur, ^2^ Mikhail V. Smelov^2^, Nina D. Ivanova^3^ and Vadim S. Ziborov^1,4^

^1^Institute of Biomedical Chemistry, Pogodinskaya str., 10, Moscow 119121, Russia

^2^Foundation of Perspective Technologies and Novations, Moscow 115682, Russia

^3^Skryabin Moscow State Academy of Veterinary Medicine and Biotechnology, Moscow 109472, Russia

^4^Joint Institute for High Temperatures of the Russian Academy of Sciences, Moscow 125412, Russia

Corresponding author: Professor Yuri D. Ivanov, e-mail: [yurii.ivanov.nata@gmail.com](mailto:yurii.ivanov.nata@gmail.com)

**Results of AFM visualization of protein adsorbed onto mica substrates from HRP solution with a concentration from 10^-9^ to 10^-6^ М**

To perform this experimental series, the samples were prepared as described in the *Methods* section of the main text (see *AFM experiments and sample preparation*). The protein solutions were prepared from the initial 10 μM solution of HRP in deionized ultrapure water by sequential tenfold dilution with ultrapure water; after mixing of the solution to be diluted with water, each solution was incubated in a shaker for 30 min. The initial 10 μM HRP solution was prepared by dissolving a certain amount of the solid commercial HRP preparation in ultrapure water.

The results of AFM visualization of the substrate surface, obtained after adsorption of the protein onto mica substrates, are presented below in Figs. S1-S4. These Figures display the images obtained by the simultaneous recording of the signal from two channels: height signal and phase signal. Also, to make the images more obvious, topography images in a more contrasting colour palette, are presented. To provide easier comparison of the images with each other, for all topography images, the Z scale was set in the range from 0 to 1.8 nm. To estimate the height of objects registered on the surface, cross-sections, corresponding to the lines on the topography images, are also presented (panel d).

As indicated in the main text, AFM visualized objects with a height of (1.2 ± 0.2) nm can be attributed to HRP molecules, adsorbed from the solution onto the substrate surface. According to Davies et al., the molecular weight of HRP is *M_r_* = 40 kDa [1]. Furthermore, other globular proteins with similar *M_r_* were reported to have comparable sizes (putidaredoxin reductase, *h_max_*=1.8 nm [2]; adrenodoxin reductase, *h_max_*=1.8 nm [3], *M_r_*=54 kDa [4]). For these considerations, one can conclude that the objects with ~1.2-nm height, observed upon AFM scanning, can be attributed to HRP monomers.

In Figs. S1-S4 (panel (с)), the objects with a height exceeding 1 nm are coloured in red. These objects are compact, and one can point out that the number of these objects in the case with 10^-6^ M HRP solution is much greater than that in the case with 10^-7^ M one. At that, such objects were virtually not observed in the analysis of 10^-8^ M and 10^-9^ M HRP solutions. Thus, one can state the number of adsorbed HRP macromolecules to be dependent on the HRP solution concentration.

In Figs. S1-S4 (panel (с)), one can also point out objects with a height from 0.3 to 0.8 nm (coloured in green). These objects are not compact, but they are flat and of various lateral sizes, what can be clearly seen in Figs. S2(с) and S3(c). So, in Fig. S2(с) one can see that the lateral sizes of the «green» objects are very different, while their height remains the same in the case of 10^-6^ M, and in the case of 10^-8^ M HRP solution. Flat structures are supposed to be contaminants — for instance, salt. Possibly, protein molecules can be present among the objects with <1 nm height, but by height they are indistinguishable from the contaminants and are thus not taken into account in our calculations.

In this way, upon processing AFM data, no less than 10 scans of different surface patches are obtained and no less than 200 objects are registered. Then, using specialized AFM data processing software, objects with height ≥1 nm are selected, and the density function of protein distribution with height *ρ(h)* is plotted. The results of AFM visualization of HRP protein, adsorbed onto mica from its solutions with a concentration from 10^-9^ M to 10^-6^ М, have indicated that the concentration at the level of 10^-7^ М is optimal, since in such a case the compact objects are located on the substrate surface separately from each other, what allows us to precisely determine their heights by AFM. This is the very concentration we used in our experiments on the study of KEMF effect on the protein, and the results obtained in these experiments are presented in the main text. At higher concentration of 10^-6^М, the height of the AFM visualized objects remains approximately the same, but the compact objects are located close to each other, and this hinders their counting.

| 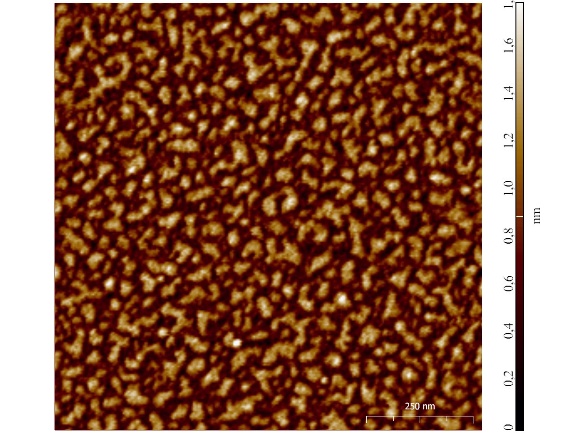(а) | 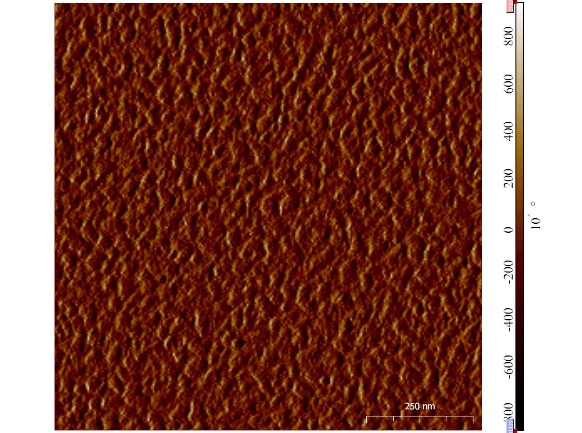(b) |
| --- | --- |
| 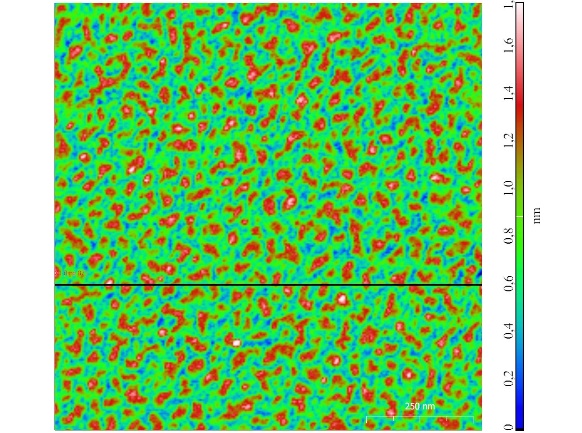(с) | 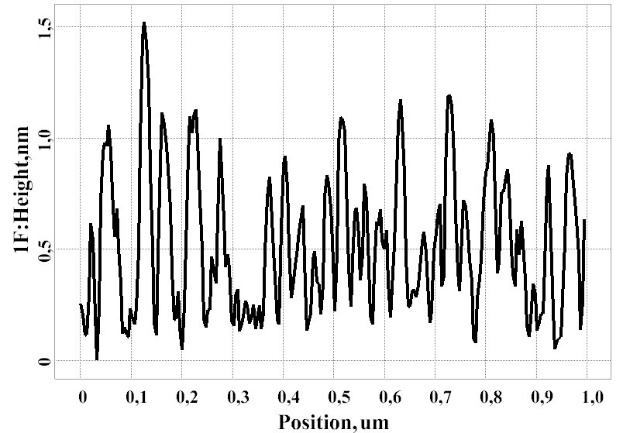(d) |
| **Fig. S1.** Results of AFM analysis of **10^-6^M** HRP solution. Height (a) and phase (b) AFM images of mica surface with adsorbed HRP macromolecules. AFM image with a more contrasting palette (c) and cross-section profile corresponding to the line in the image in c (d). Scan size 1×1 µm^2^, 256×256 points, Z scale (a, c) from 0 to 1.8nm. | |

| 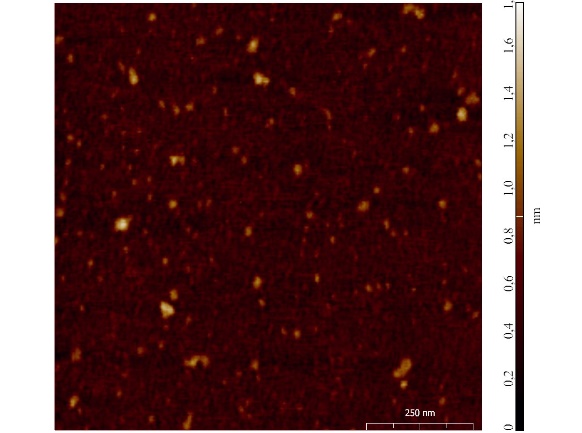(a) | 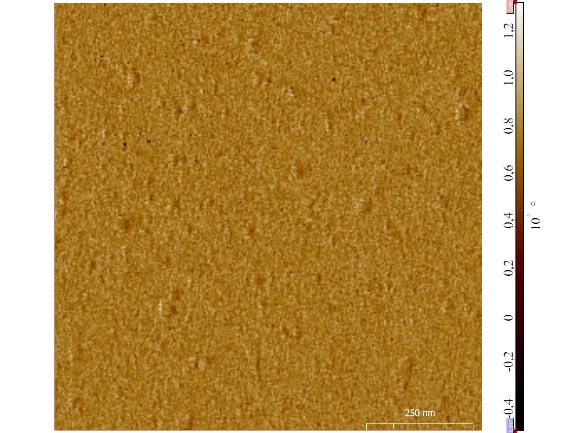(b) |
| --- | --- |
| 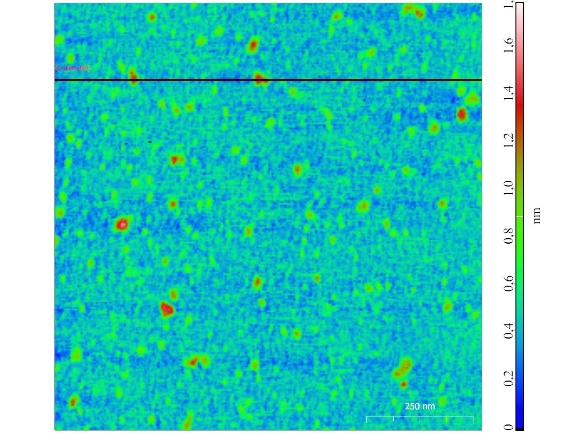(с) | 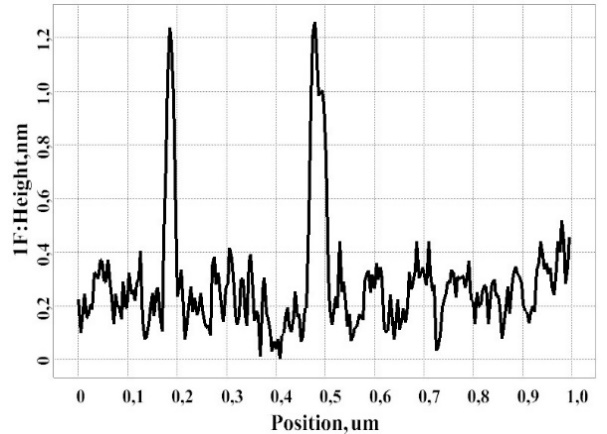(d) |
| 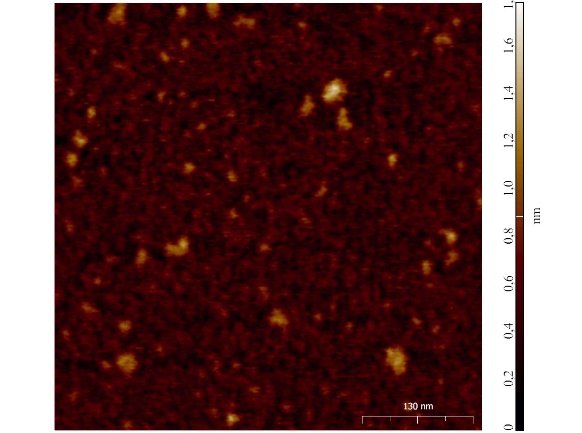(е) | 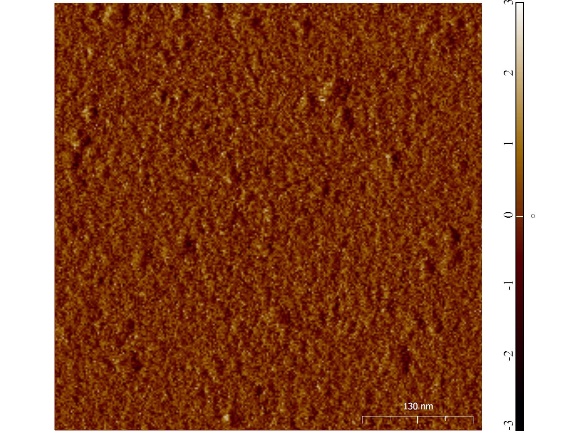(f) |
| **Fig. S2.** Results of AFM analysis of **10^-7^ M** HRP solution. Height (a, c, e) and phase (b, f) AFM images of mica surface with adsorbed HRP macromolecules. AFM image with a more contrasting palette (c) and cross-section profile corresponding to the line in the image in c (d). Scan size 1×1 µm^2^ (a, c) and 0.5×0.5 µm^2^ (e), 256×256 points, Z scale (a, c, e) from 0 to 1.8nm. | |

| 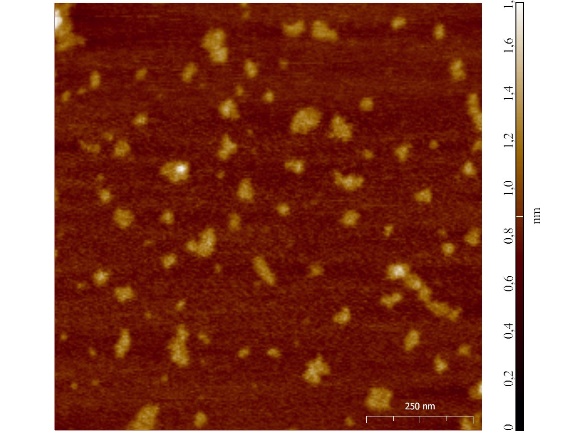(a) | 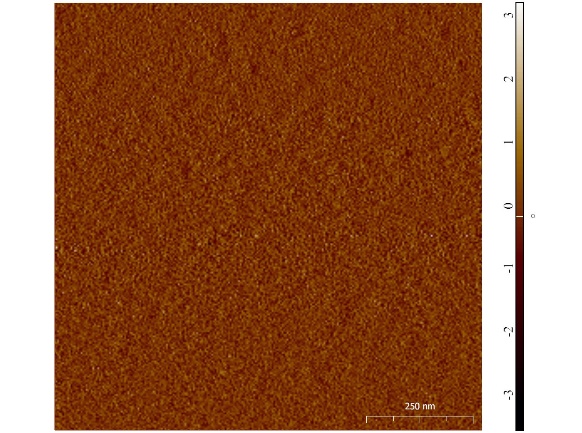(b) |
| --- | --- |
| 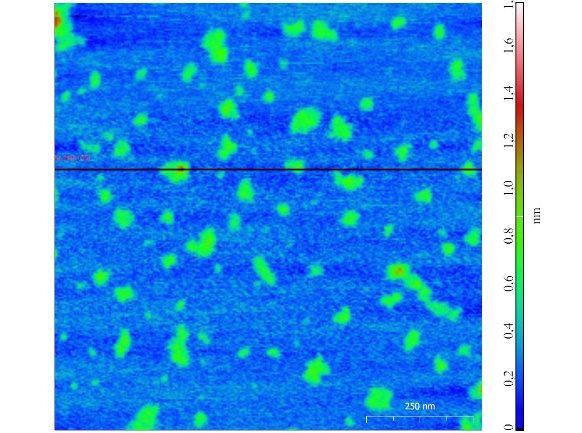(c) | 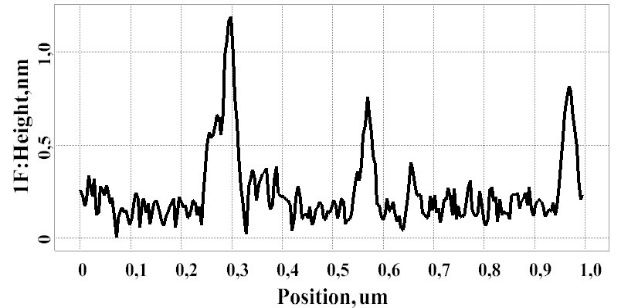(d) |
| **Fig. S3.** Results of AFM analysis of **10^-8^ M** HRP solution. Height (a) and phase (b) AFM images of mica surface with adsorbed HRP macromolecules. AFM image with a more contrasting palette (c) and cross-section profile corresponding to the line in the image in c (d). Scan size 1×1 µm^2^, 256×256 points, Z scale (a, c) from 0 to 1.8 nm. Arrows indicate typical objects with heights from 0.3 to 0.8 nm and small (white arrows) or large (black arrows) lateral sizes. | |

| 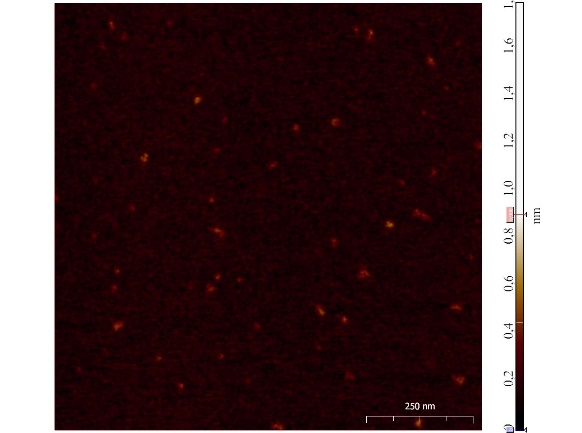(a) | 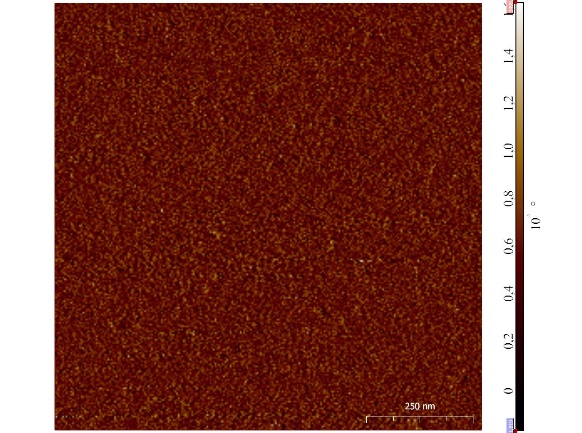(b) |
| --- | --- |
| 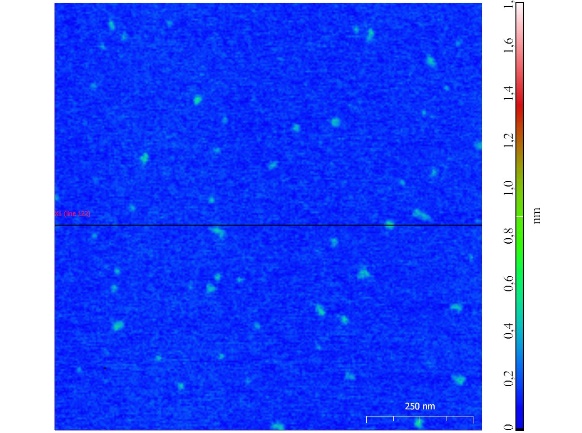(c) | 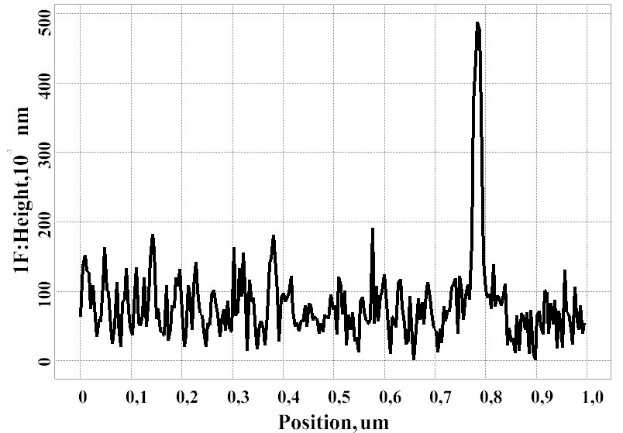(d) |
| **Fig. S4.** Results of AFM analysis of **10^-9^ M** HRP solution. Height (a) and phase (b) AFM images of mica surface with adsorbed HRP macromolecules. AFM image with a more contrasting palette (c), and cross-section profile corresponding to the line in the image in c (d). Scan size 1×1 µm^2^, 256×256 points, Z scale (a, c) from 0 to 1.8nm. The Y axis scale is given in ×10^-3^nm units. | |

**References**

1. Davies, P. F., Rennke, H. G., Cotran, R. S. Influence of molecular charge upon the endocytosis and intracellular fate of peroxidase activity in cultured arterial endothelium. J. Cell Sci. **49** (1), 69-86 (1981).
2. Ivanov, Y. D. et al. AFM study of cytochrome CYP102A1 oligomeric state. Soft Matter **8** (17), 4602-4608 (2012).
3. Ivanov, Y. D., Frantsuzov, P. A., Zöllner, A., et al. Atomic force microscopy study of protein–protein interactions in the cytochrome CYP11A1 (P450scc)-containing steroid hydroxylase system. Nanoscale Res. Lett. **6** (1), 54; 10.1007/s11671-010-9809-5 (2011).
4. Chu, J. W., & Kimura, T. Studies on Adrenal Steroid Hydroxylases Molecular and catalytic properties of adrenodoxin reductase (a flavoprotein). J. Biol. Chem. **248** (6), 2089-2094 (1973).
